# METTL3 attenuates ferroptosis sensitivity in lung cancer via modulating TFRC

**DOI:** 10.1515/med-2023-0882

**Published:** 2024-01-10

**Authors:** Peng Zhang, Su Wang, Yuanyuan Chen, Qingbo Yang, Jian Zhou, Wangfu Zang

**Affiliations:** Department of Cardio-Thoracic Surgery, Shanghai Tenth People’s Hospital, School of Clinical Medicine of Nanjing Medical University, Shanghai 200072, China; Department of Cardio-Thoracic Surgery, Shanghai Tenth People’s Hospital Affiliated to Tongji University, Shanghai 200072, China; Department of Cardio-Thoracic Surgery, Shanghai Tenth People’s Hospital, School of Clinical Medicine of Nanjing Medical University, No. 301 Yanchang Middle Road, Jing'an District Shanghai 200072, China

**Keywords:** lung cancer, ferroptosis, METTL3, TFRC

## Abstract

Overexpression of methyltransferase-like 3 (METTL3) is significantly correlated with the malignancy of lung cancer (LC). In the present study, we demonstrated that METTL3 had higher levels in LC tissues relative to normal tissues. METTL3 showed superior sensitivity and specificity for diagnosis and identification of LC functions. In addition, silencing METTL3 resulted in enhanced ferroptosis sensitivity, whereas overexpression of METTL3 exhibited the opposite effect. Inhibition of METTL3 impeded LC growth in cell-derived xenografts. Further exploratory studies found that METTL3 stimulated the low expression of transferrin receptor (TFRC), which was critical for ferroptosis sensitization in LC cells induced by silenced METTL3, as silencing of TFRC caused a decrease in negative regulators of ferroptosis (FTH1 and FTL) in METTL3 knockdown A549 and PC9 cells. Finally, we confirmed that METTL3 attenuation effectively maintained the stability of TFRC mRNA. In conclusion, we reported a novel mechanism of METTL3 desensitization to ferroptosis via regulating TFRC, and an appropriate reduction of METTL3 might sensitize cancer cells to ferroptosis-based therapy.

## 1 Introduction

Lung cancer (LC) accounts for 11.4% of malignancies and has leapt to the top of the list of cancer “super killers.” In particular, the prevalence of LC varies considerably by country and gender [1,2]. Men are more likely to develop LC than women, and the incidence and mortality ratios are 2:1 for both sexes. The occurrence of LC is age-related, mostly in the middle-aged and elderly population (45–65 years old), but in recent years there has been a trend toward younger age [3]. Indeed, LC exhibits rapid proliferation, metastasis, and intertumoral heterogeneity, resulting in poor patient survival due to frequent tumor recurrence and metastasis, discouraging overall survival [4]. Therefore, a more accurate understanding of the molecular mechanisms of LC, identification of new diagnostic molecular markers and new therapeutic modalities could help to detect LC at an early stage and develop appropriate treatment options for tumor progression.

Multicellular organisms possess the ability to remove damaged or redundant cells to maintain normal development of the organism and prevent the development of hyperproliferative diseases [5]. Thus, regulated cell death (RCD), such as apoptosis and necrosis, occurs in normal physiological programs [6]. However, either excessive or failing RCD can be a causative agent of cancer, especially in the lung [7]. In contrast to the known forms of cell death, ferroptosis was thought to be a new form of iron-dependent RCD, whose key feature is the lethal accumulation of iron-dependent reactive oxygen species (ROS) and lipid peroxides [8]. Several known molecules (e.g., glutathione peroxidase 4, p53, heat shock protein β-1, and nuclear factor E2-related factor 2) target iron metabolism or lipid peroxidation by specific mechanisms to alter ferroptosis sensitivity [9]. Furthermore, enhanced intracellular iron load is required to maintain ferroptosis [10]. More direct evidence came from the fact that suppression of the iron metabolism transcription factor IREB2 increased the expression of iron metabolism-related proteins (FTH1 and FTL), and that raised iron storage capacity effectively aggravated ferroptosis resistance [11]. In recent years, another new key player in the ferroptosis pathway, methyltransferase 3 (METTL3), has emerged. In-depth studies have revealed that METTL3 strengthened the expression of SLC7A11, which is a crucial negative regulator of ferroptosis, by means of its N6-methyladenosine (m^6^A) activity in hepatoblastoma and LC. Thus, in turn, it led to enhanced resistance of tumor cells to ferroptosis and induced malignant progression of cancer [12,13]. The hindrance of ferroptosis by METTL3 appeared to be dependent on the m^6^A pathway, both in tumors and in other diseases, such as brain hemorrhage [14] and diabetes mellitus [15]. However, regulatory mechanism of METTL3 on ferroptosis is complex and multifactorial and still requires in-depth investigation.

In this study, we reported heightened expression of METTL3, a common tumorigenic event in LC. Besides, we found an important role of METTL3 in mediating cellular ferroptosis sensitivity, i.e., ablation of METTL3 caused greatly enhanced LC cellular ferroptosis sensitivity. Also, we identified the negative regulation of transferrin receptor (TFRC) by METTL3 and the desensitization of cells to ferroptosis induced by TFRC attenuation. These results suggested that METTL3 combined with TFRC to induce ferroptosis could be used as a promising cancer therapy to address LC.

## 2 Materials and methods

### 2.1 LC tissue samples and cell cultures

A total of 40 pairs of tumor samples (LC specimens and paired adjacent normal tissue specimens) removed during surgery were obtained from Shanghai Tenth People’s Hospital, School of Clinical Medicine of Nanjing Medical University for this study. Patients with a history of anti-tumor therapy and those with other diseases were not included in this study. The protocol for the use and collection of human tissues was reviewed and approved by the Shanghai Tenth People’s Hospital, School of Clinical Medicine of Nanjing Medical University Ethics Committee. All participants were carefully informed and signed a written consent form before being subjected to the study. All experiments were performed in strict accordance with relevant guidelines and regulations and in compliance with the *Declaration of Helsinki*.

We obtained the normal human bronchial epithelial cell line NL20 and four human typical LC cell lines (A549, H1299, PC9, H1975, and HCC827) from the Cell Bank of the Chinese Academy of Sciences (Shanghai, China) for this study. All LC cell lines and NL20 cells were preserved in penicillin/streptomycin solution (1%, Gibco, USA) and 10% FBS (Sagecreation, China) enhanced with commercially available 1640 medium (Gibco, USA) according to the manufacturer’s requirements. All culture flasks were placed in humid air (culture environment: 37°C, 5% CO_2_) to maintain cell growth.

### 2.2 Plasmid construction and cell transfection

Specific small interfering RNAs (si-NC: CATTGACTAAGCCGATAGCCCGACTAGCT, si-TFRC 1#: GCGGTTCTTGGTACCAGCAACTTCA, si-TFRC 2#: CAGCCCACTGTTGTATACGCTTATT, si-METTL3 1#: GGAGGAGTGCATGAAAGCCAGTGAT, and si-METTL3 2#: AGCTGCACTTCAGACGAATTATCAA) targeting specific regions of METTL3 and TFRC were constructed and obtained by Obio Technology (Shanghai, China), and next, the sequences with the highest knock-down efficiency were selected for subsequent studies. Overexpression of intracellular METTL3 was achieved by ordering an overexpression plasmid (GV144 and oe-METTL3) from GeneChem (Shanghai, China), and an empty plasmid vector (oe-NC) served as a control. Transfection of METTL3 overexpressing plasmids and siRNAs targeting specific genes was achieved in A549 and PC9 cells with the aid of Lipofectamine 3000 (Invitrogen, USA) according to the manufacturer’s requirements.

### 2.3 *In vitro* cell viability assay

The viability values of two LC cell lines (A549 and PC9) after different treatments were determined using the Cell Counting Kit-8 (CCK-8, RiboBio, China) method. The prepared suspensions of multiple groups of target cells were sequentially injected into 96-well plates (5 × 10^3^ cells/well). At the specified time points (0, 24, 48, 96 h), 10 μL of CCK-8 reaction reagent (ApexBio, USA) was added to the cell cultures to continue maintenance for 2 h (37°C, dark environment). Finally, the optical density (OD) values of each well were recorded at 450 nm using an automatic microplate reader (Tecan M200 PRO, Belgium).

### 2.4 4′-6-Diamidino-2-phenylindole (DAPI)/propidium iodide (PI) double staining

A549 and PC9 cells were treated with si-NC or si-METTL3 in 12-well plates (2 × 10^5^ cells/well). To detect cell death, cells were stained with PI for 5 min in the dark and then restained with DAPI. Cells were washed three times in PBS to remove excess dye and then photographed with a fluorescence microscope (Olympus, BX53). PI staining was repeated in triplicate to obtain accurate results.

### 2.5 RNA stability assay

A549 cells and PC9 cells were treated with siRNA against METTL3. After 48 h, METTL3 knockdown or non-knockdown cells were mixed with actinomycin D (final concentration 5 μg/mL, APExBIO) to block the synthesis of new RNA in the cells. Next, RNA was extracted and collected at the indicated time points (1, 6, 12, 18, and 24 h) to perform RT-qPCR for stability assessment of TFRC mRNA.

### 2.6 Determination of intracellular lipid ROS, malondialdehyde (MDA), and glutathione (GSH) content

The levels of lipid ROS in the target cells were examined with the aid of a fluorescent probe C11-BODIPY 581/591 (5 μmmol, Thermo Fisher), which was sensitive to lipid peroxidation. A549 and PC9 cells were treated as required, followed by digesting (0.25% trypsin), washing, and resuspending cells in fresh medium supplemented with 10% FBS. Cells were stained with C11-BODIPY for 30 min under the prescribed conditions (protected from light, 37°C). Finally, the stained cells were collected for flow cytometry (BD Accuri C6) to collect the mean fluorescence intensity of lipid ROS-positive LC cells.

LC intracellular MDA levels were determined using a MDA assay kit (Abcam) according to the manufacturer’s standard protocol. Importantly, the complete MDA level was normalized to the total protein content of the sample and recorded as nmol/mg.

Total GSH content in tumor cells (50,000/group) was calculated by ordering a commercial GSH assay kit from Beyotime Biotechnology. The concentration of GSH was normalized according to the total protein content of the treated samples and denoted as nmol/mg.

### 2.7 Analysis of intracellular unstable iron content

Supernatants collected from LC cells of groups si-NC, oe-NC, si-METTL3, oe-METTL3, si-METTL3 + si-NC, and si-METTL3 + si-TFRC were analyzed for unstable iron (Fe^2+^) levels. Briefly, Iron Detection Kit was purchased from Abcam, and 5 µL of iron buffer was added to each sample well and incubated at 37°C for 30 min. Iron probe of 100 µL was continued to be added to the mixture awaiting detection, mixed well and maintained for 60 min (37°C, protected from light). Immediately after completion of the reaction, the OD at 593 nm was evaluated on a micro-plate reader.

### 2.8 Immunohistochemistry (IHC)

The strong and weak distribution of METTL3 expression in LC tumor tissues was identified by IHC. Tumor samples were fixed in tissue fixative (4% paraformaldehyde) and prepared into 4 μm paraffin sections with the addition of 3% H_2_O_2_ to block endogenous peroxidase activity. Antigen retrieval was done in 10 mM citrate solution. Sections were soaked with diluted primary antibody targeting METTL3 (1:100, Abcam, UK) in a humidified refrigerator at 4°C for 12 h. Sections were then rinsed with PBS and maintained for a further 60 min at 37°C with secondary antibody (Dako). Sections were developed with diaminobenzidine (DAB, Gene Tech) after re-rinsing with PBS, and the degree of METTL3 enrichment in the tumor tissue was determined by the intensity of staining.

### 2.9 M^6^A-RNA immunoprecipitation (Me-RIP) assay

A549 and PC9 cells transfected with si-NC or si-METTL3 were lysed, and the clarified lysates were subsequently incubated with appropriate amounts of the corresponding antibodies (anti-m^6^A, 1:500, Abcam, USA) as well as human IgG-conjugated magnetic beads in RIP buffer until overnight. The resulting magnetic bead–antibody complexes were resuspended and digested with proteinase K buffer to isolate and extract the immunoprecipitated target RNA. Purified RNA was quantified by RT-qPCR to verify the relative levels of TFRC.

### 2.10 RT-qPCR

Total RNA was isolated from LC tissue specimens, A549 and PC9 cells using RNA extraction reagent (TRIzol, Invitrogen, USA). The purity and concentration of the obtained total RNA were determined, and then the generation of cDNA templates was initiated using the HiFiScript cDNA synthesis kit (CWBIO, China). Next, PCR kits with specific primers (SYBR Green Master Mix, Applied Biosystems) and QuantStudio 3D system (Thermo Fisher, USA) were employed to complete the remaining RT-qPCR steps. The levels of both METTL3 and TFRC were fully normalized to β-actin. The relative expression levels of target genes in treated cells were calculated by the 2^–∆∆Ct^ method. The primer sequences of the specific genes used were:

METTL3-F: 5′-AAGCTGCACTTCAGACGAAT-3′

METTL3-R: 5′-GGAATCACCTCCGACACTC-3′

TFRC-F: 5′-ACCTGTCCAGACAATCTCCAG-3′

TFRC-R: 5′-TGTTTTCCAGTCAGAGGGACA-3′

β-Actin-F: 5′-TGAGAGGGAAATCGTGCGTGAC-3′

β-Actin-R: 5′-AAGAAGGAAGGCTGGAAAAGAG-3′

### 2.11 Western blot

A549 and PC9 cells were harvested and lysed in lysis buffer (Beyotime) supplemented with a 1% protease inhibitor (Roche, USA) mixture and whole proteins were extracted. Protein concentrations were estimated using the BCA Protein Assay Kit (Pierce, USA). Equal amounts of denatured protein samples were resolved on 10–12% SDS-PAGE and transferred to PVDF (Millipore, USA) membranes. Membranes were sealed with 5% skim milk for 2 h and then incubated overnight with diluted primary antibodies specific for METTL3 (1:200, Abcam), TFRC (1:1,000, Abcam), FTH1 (1:500, ABclonal), FTL (1:250, Abcam), and β-actin (1:1,000, CST) at 4°C. The membranes were continued to be incubated with secondary antibodies for 1.5 h before visualization of protein blot images using the ECL kit (Millipore, USA).

### 2.12 Animal studies

Stable knockdown of METTL3 was achieved by transfection of A549 cells with shRNA specifically targeting METTL3. Approximately 5 × 10^6^ A549 cells (0.1 mL) were injected subcutaneously into the axillae of male athymic BALB/C nude mice (4–6 weeks old, 18–22 g, 3 per group). The length (*L*) and width (*W*) of the tumors were measured weekly using calipers, and the tumor volume was calculated according to the formula *V* = (*W* ^2^ × *L*)/2. All mice were sacrificed 4 weeks later, and tumor tissues were isolated and examined for weight.

### 2.13 Statistical analysis

The experiments used were performed at least three times independently and the data obtained are reported as mean ± SD. Statistical differences between two or more consecutive data groups were compared using Student’s *t*-test and one-way analysis of variance. *Pearson* linear correlation analysis was applied to calculate the correlation between METTL3 and TFRC expression. Graphs were plotted by GraphPad Prism 8.0 software. *P* values less than 0.05 were determined to be statistically significant.

## 3 Results

### 3.1 LC is associated with up-regulation of METTL3

**Figure 1:**
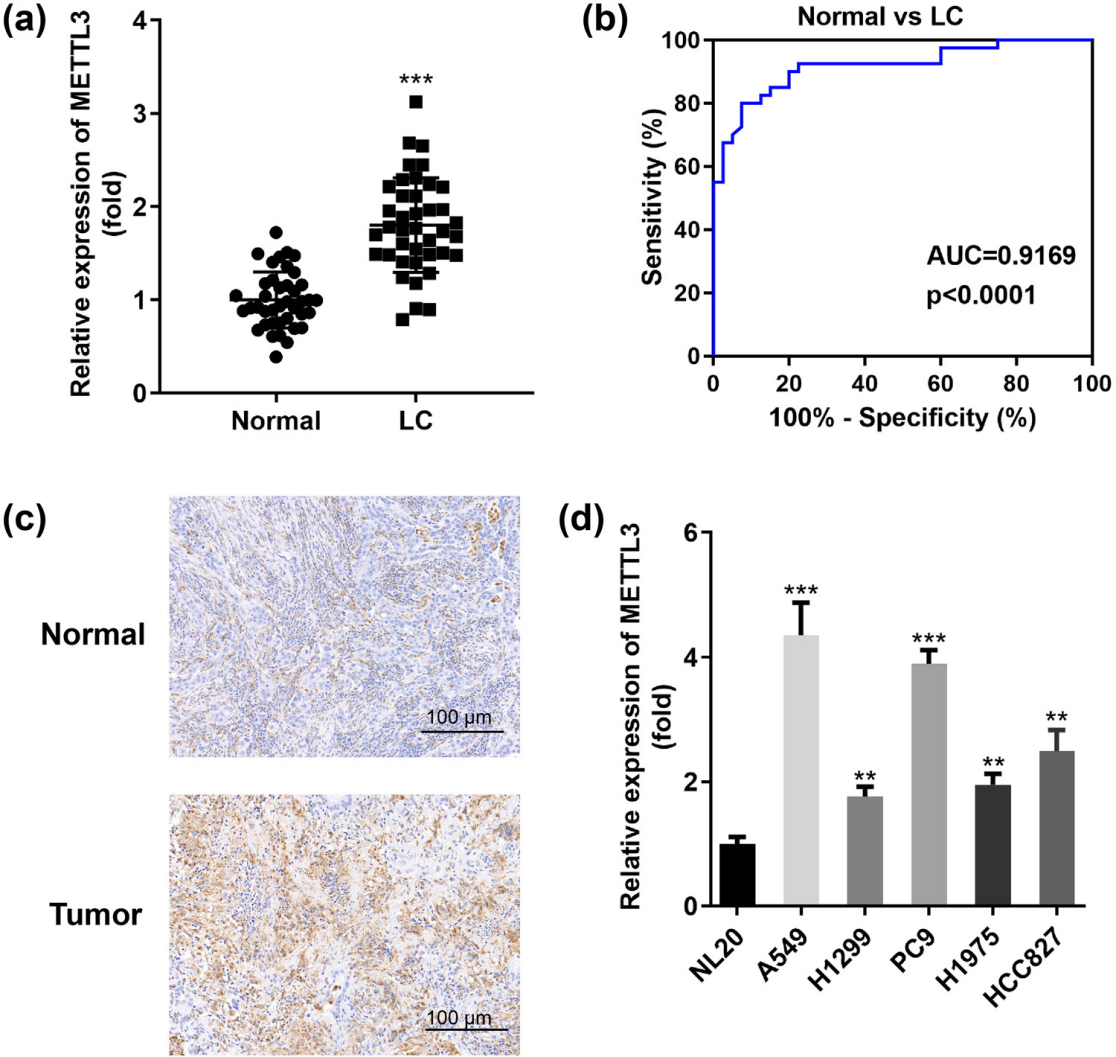
METTL3 exists in LC tumors in an overexpression pattern and is a promising biomarker for LC patients. (a) RT-qPCR assay was applied to show METTL3 expression in 40 pairs of collected clinical LC tumor tissues and paired normal tissues (Normal). (b) ROC curves were established to assess the potential diagnostic value of METTL3 based on its expression in tumor samples. (c) Typical IHC images of METTL3 staining in paired LC tumor tissues. Scale bar, 100 μm. (d) Differences in METTL3 expression levels between human bronchial epithelial cells (NL20) and representative LC cells (A549, H1299, PC9, H1975, and HCC827). **P < 0.01, ***P < 0.001.

Considering the important role of METTL3 in controlling the growth, survival, and invasion of LC cells (A549, H1299) [16], we were motivated to explore additional mechanisms of METTL3 in LC. In the current study, we described and analyzed METTL3 expression levels in a cohort of LC patients and compared them with normal paracancer controls. Tissues from patients with untreated primary LC showed a strong up-regulation of METTL3 (Figure 1a). To further investigate the clinical significance of METTL3 in LC, we preprocessed the clinical data from archived LC patients and plotted ROC curves. As shown in Figure 1b, METTL3 presented sensitivity and specificity in distinguishing between healthy individuals and patients with an AUC of 0.9169 (*P* < 0.0001), indicating the strong detection ability of METTL3 in the early diagnosis of LC. In addition, evaluation of stored tissue samples by IHC staining showed a substantial increase in staining for METTL3 in lung tumors, which was consistent with previous observations of mRNA in tissues (Figure 1c). Indeed, relatively high levels of METTL3 were detected in five LC cell lines (A549, H1299, PC9, H1975, and HCC827). Among them, we noted that METTL3 was most significantly upregulated in A549 and PC9 cells compared to other types of LC cells, and we used these two cells as subjects for our next study (Figure 1d). In conclusion, these data suggested that METTL3 was indeed increased in LC and its presence might serve as a potential marker for early diagnosis in LC patients.

### 3.2 METTL3 ablation activates ferroptosis in LC cells

**Figure 2:**
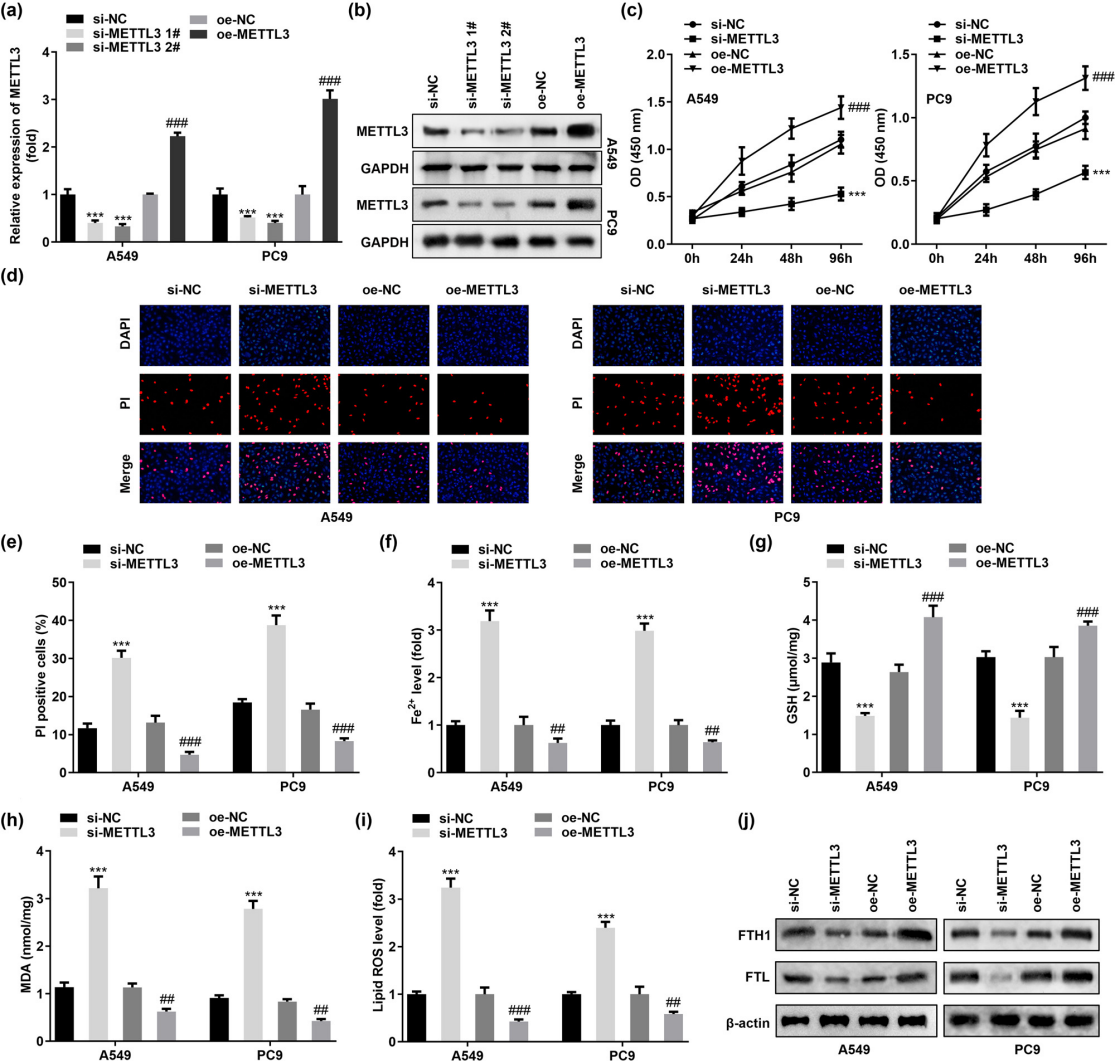
METTL3 silencing is critical for the induction of ferroptosis sensitivity in LC cells. (a and b) Knockdown or overexpression efficiency of METTL3 was verified by RT-qPCR and western blot after introduction of METTL3 siRNAs (si-METTL3 1# and si-METTL3 2#) and METTL3 overexpression plasmid (oe-METTL3) in A549 and PC9 cells, respectively. Among them, si-METTL3 2# showed stronger knockdown efficiency. (c) CCK-8 assay was conducted to obtain data on the change in cell viability after overexpression or knockdown of METTL3 in A549 and PC9 cells, respectively. (d and e) DAPI/PI staining was performed to detect cell death. (f–i) Determination of Fe^2+^, GSH, MDA, and lipid ROS in METTL3 knockdown or overexpression cells. (j) Expression of FTH1 and FTL proteins, the negative regulators of ferroptosis, was detected by western blot. ^##^ *P* < 0.01, ^###^ *P* < 0.001, ***P < 0.001.

To continue to systematically investigate the potential role of METTL3 in LC, we successfully constructed knockdown and overexpression of METTL3 at the cellular level by transfecting two human LC cell lines, A549 and PC9, with two independent siRNAs (named si-METTL3 1# and si-METTL3 2#) and METTL3 overexpression plasmids (named oe-METTL3), respectively (Figure 2a and b). As expected, ablation of METTL3 inhibited the cell viability of A549 and PC9, while enhanced METTL3 promoted LC cell growth *in vitro* (Figure 2c). Subsequently, we performed DAPI/PI double staining to evaluate cell death. DAPI stained nuclei (bright blue fluorescence) and PI stained dead cells (red fluorescence). The results showed that METTL3 knockdown caused an increased rate of PI-positive cells, suggesting increased cell death. In contrast, PI-positive cells were decreased by METTL3 overexpression (Figure 2d and e). The above data linked METTL3 to cell death. Ferroptosis was a newly identified form of cell death, and METTL3 has recently been found to cause altered ferroptosis sensitivity in LC cells [12]. Therefore, we sought to explore in A549 and PC9 cells whether METTL3 levels determine the degree of sensitivity of tumor cells to ferroptosis. Redox-active iron enrichment, GSH depletion, and lipid ROS accumulation were recognized as central elements of ferroptosis. Therefore, we monitored the levels of Fe^2+^, GSH, oxidative stress markers MDA, and lipid ROS in target cells. Our results presented that silencing METTL3 resulted in a decrease in GSH concentration and an increase in Fe^2+^ level, MDA content, and lipid ROS level in A549 and PC9 cells. Importantly, the opposite phenomenon was presented in A549 and PC9 cells overexpressing METTL3 (Figure 2f–i). Furthermore, we found that METTL3 silencing significantly hindered the expression of negative regulators of ferroptosis (FTH1 and FTL) in cells, and conversely, the levels of FTH1 and FTL proteins increased in METTL3 overexpressing cells (Figure 2j). The above convincing data strongly demonstrated that METTL3 assumed an important role in ferroptosis in LC cells, and that inhibition of METTL3 induced stronger ferroptosis sensitivity in LC cells.

**Figure 3:**
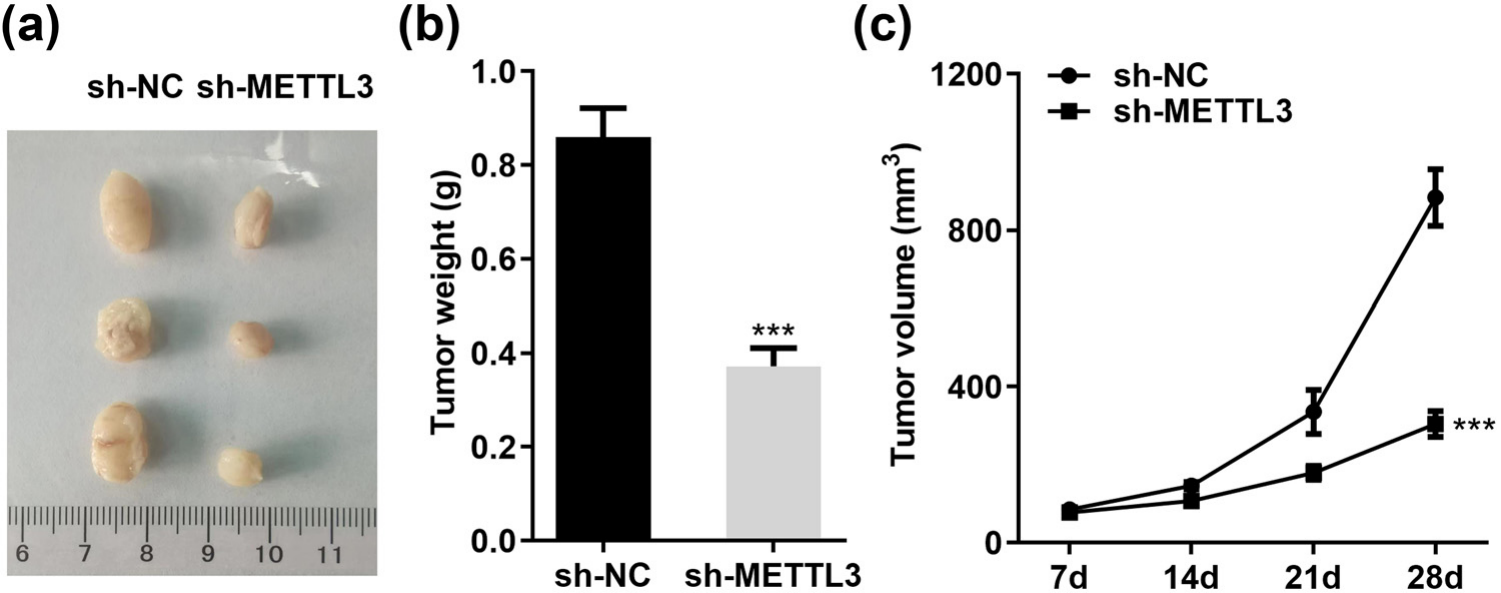
METTL3 accelerates the growth of LC cells *in vivo*. (a) Pictures of xenografts isolated from nude mice after sacrifice. (b and c) Tumor weights (b) and growth curves (c) of subcutaneous xenograft models injected with stably knockdown METTL3 (sh-METTL3) and negative control (sh-NC) A549 cells. ***P < 0.001.

Next, we performed subcutaneous xenograft experiments to assess the physiological relevance of METTL3 to LC cell growth *in vivo*. Consistent with the *in vitro* results, tumor growth was slowed and xenograft weight was reduced when implanted with METTL3 knockdown A549 cells relative to control (sh-NC) cells (Figure 3a–c). Collectively, these loss/gain-of-function experiments confirmed that METTL3 promoted LC cell growth and proliferation both *in vivo* and *in vitro*.

### 3.3 METTL3 triggers TFRC low expression in LC cells

**Figure 4:**
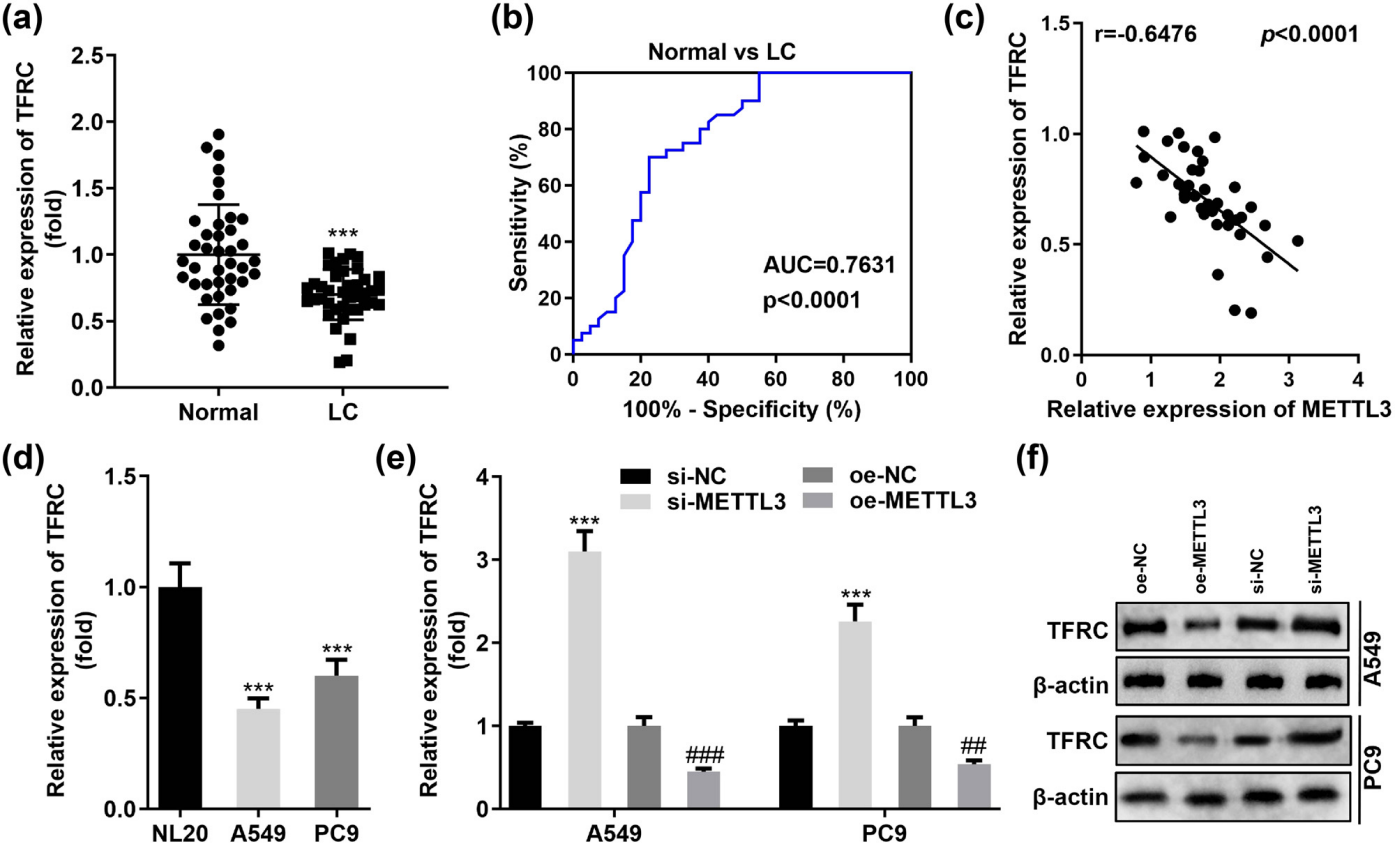
METTL3 negatively regulates TFRC levels in LC. (a) TFRC was significantly reduced in 40 primary LC tissues using normal tissues adjacent to the cancer as controls. (b) ROC curves were established to examine the practical value of TFRC in clinical practice. (c) Correlation between METTL3 and TFRC was analyzed based on RT-qPCR data. (d) Restricted TFRC levels were found in two representative LC cell lines (A549 and PC9). (e and f) Relative levels of TFRC mRNA and protein were detected by RT-qPCR and western blotting in A549 and PC9 cells transfected with si-METTL3 and oe-METTL3, respectively. ^##^ *P* < 0.01, ^###^ *P* < 0.001, ***P < 0.001.

Ferroptosis is closely related to intracellular iron homeostasis, and TFRC is one of the essential molecules for increasing intracellular iron content as well as maintaining intracellular iron homeostasis [17]. Thus, we analyzed TFRC levels in LC specimens and normal paracancerous tissues from 40 patients. RT-qPCR results determined that TFRC was generally downregulated in LC tumor tissues (Figure 4a). The data from our cohort also showed the potential value of TFRC expression levels for the diagnosis of LC. As illustrated in Figure 4b, TFRC was sensitive and specific in distinguishing between LC patients and healthy controls with an AUC of 0.7631. Considering that TFRC was one of the important genes for intracellular iron uptake, we analyzed the correlation between the expression of TFRC and METTL3 in tissues based on the obtained data. Figure 4c indicates a significant negative correlation between TFRC and METTL3 in LC. Does the expression level of METTL3 actually determine the expression of TFRC in A549 and PC9 cells? To answer this question, we first analyzed TFRC expression in A549 and PC9 as well as NL20 cells. As expected, TFRC expression was remarkably lower in A549 and PC9 cells than in normal bronchial epithelial cells NL20 (Figure 4d). Furthermore, the expression of both TFRC mRNA and protein was enhanced in the absence of METTL3 in A549 and PC9 cells. In contrast, the introduction of METTL3 overexpression vector successfully decreased intracellular TFRC mRNA and protein levels (Figure 4e and f). Therefore, it was reasonable to speculate that METTL3 affected the sensitivity of LC cells to ferroptosis by reducing intracellular TFRC levels.

### 3.4 TFRC is critical for ferroptosis sensitivity in METTL3-silenced LC cells

**Figure 5:**
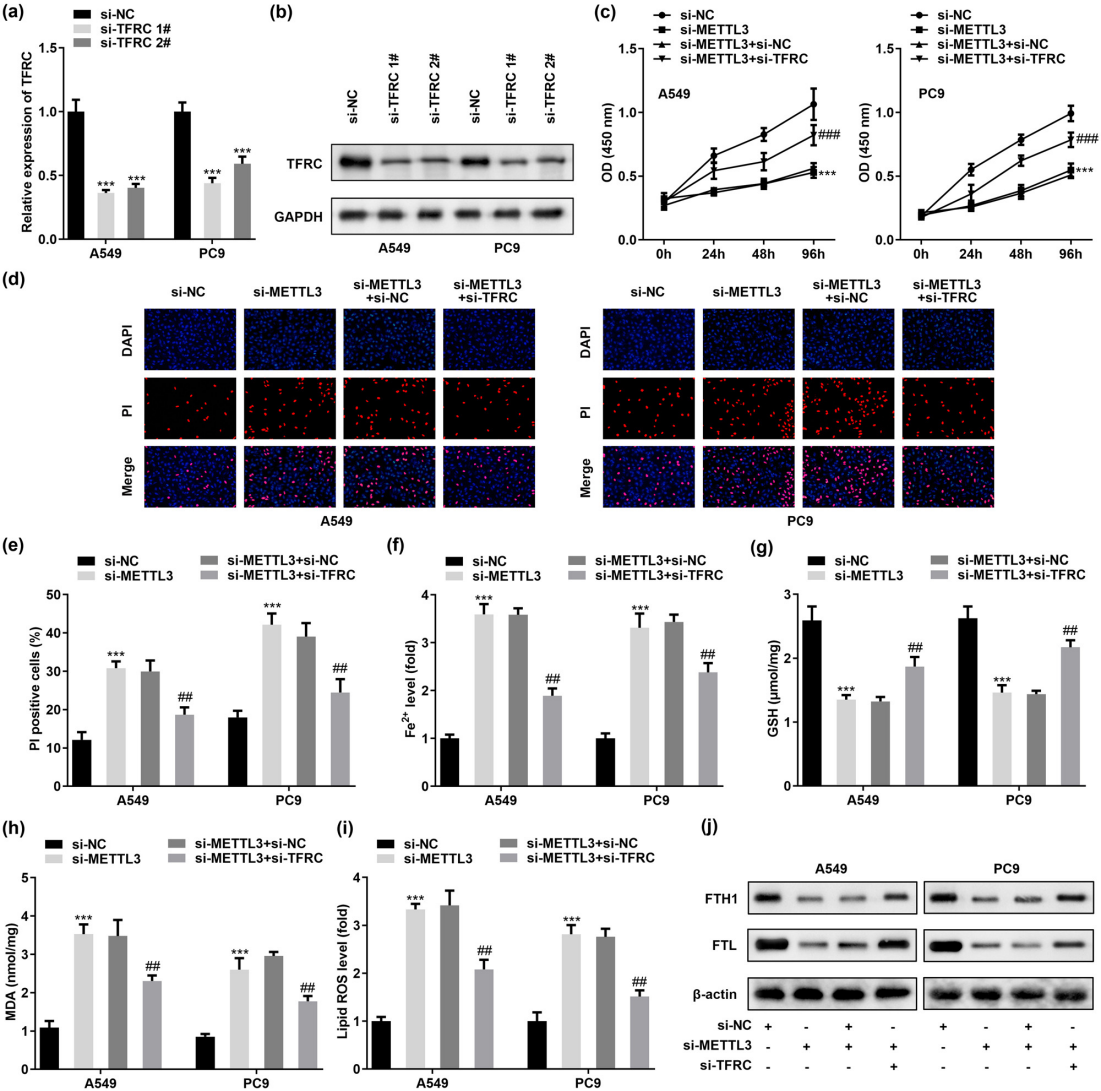
METTL3 knockdown-induced ferroptosis is limited by the decreased TFRC expression levels in LC cells. A549 and PC9 cells were transfected with si-NC, si-METTL3, si-METTL3 + si-NC, and si-METTL3 + si-TFRC, respectively. (a and b) RT-qPCR and western blot was employed to verify the knockdown efficiency of si-TFRC 1# and si-TFRC 2# in A549 and PC9 cells. (b) CCK-8 assay was performed to detect the effect of TFRC silencing on the viability of METTL3-knockdown A549 and PC9 cells. (c and d) DAPI/PI staining was used to verify the effect of TFRC silencing on the death of METTL3-knockdown A549 and PC9 cells. (f–i) Concentrations of Fe^2+^, GSH, MDA, and lipid ROS in A549 and PC9 cells were evaluated after co-transfection of si-TFRC and si-METTL3. (j) Protein expression of FTH1 and FTL in A549 and PC9 cells co-transfected with si-TFRC and si-METTL3 was measured by western blotting. ^##^ *P* < 0.01, ^###^ *P* < 0.001, ***P < 0.001.

To confirm that METTL3-blocked TFRC expression inhibited intracellular iron uptake and thus caused ferroptosis desensitization in LC cells, we first designed two siRNAs (named si-TFRC 1# and si-TFRC 2#) to deplete TFRC in A549 and PC9 cells (Figure 5a) and verified their knockdown efficiency, respectively (Figure 5b). In addition, we added si-TFRC in METTL3-silenced A549 and PC9 cells and monitored the cellular activity. The results clearly demonstrated that the decrease in cellular activity or cell death caused by METTL3 attenuation could be rescued by si-TFRC treatment (Figure 5c). In addition, si-TFRC treatment also rescued A549 and PC9 cell death induced by METTL3 deletion (Figure 5d and e). Ferroptosis is the result of lethal accumulation of unstable iron in cells, and indeed, METTL3 silencing still increased intracellular Fe^2+^ levels even though TFRC was reduced in A549 and PC9 cells. Notably, ablation of TFRC weakened this effect (Figure 5f). Similarly, we also monitored the role of TFRC in regulating GSH concentration, MDA accumulation, and lipid ROS levels. The data showed that lower levels of TFRC caused an increase in GSH concentration, a decrease in MDA accumulation, and lipid ROS levels in A549 and PC9 cells after si-METTL3 treatment (Figure 5g–i). In addition, the opposite results of FTH1 and FTL induced by METTL3 silencing were also attenuated after knocking down TFRC in A549 and PC9 cells (Figure 5j). Combining the above data, we concluded that the induction of ferroptosis in LC cells by METTL3 silencing was dependent on the level of intracellular TFRC.

### 3.5 METTL3-mediated m^6^A modification reduces TFRC stability

**Figure 6:**
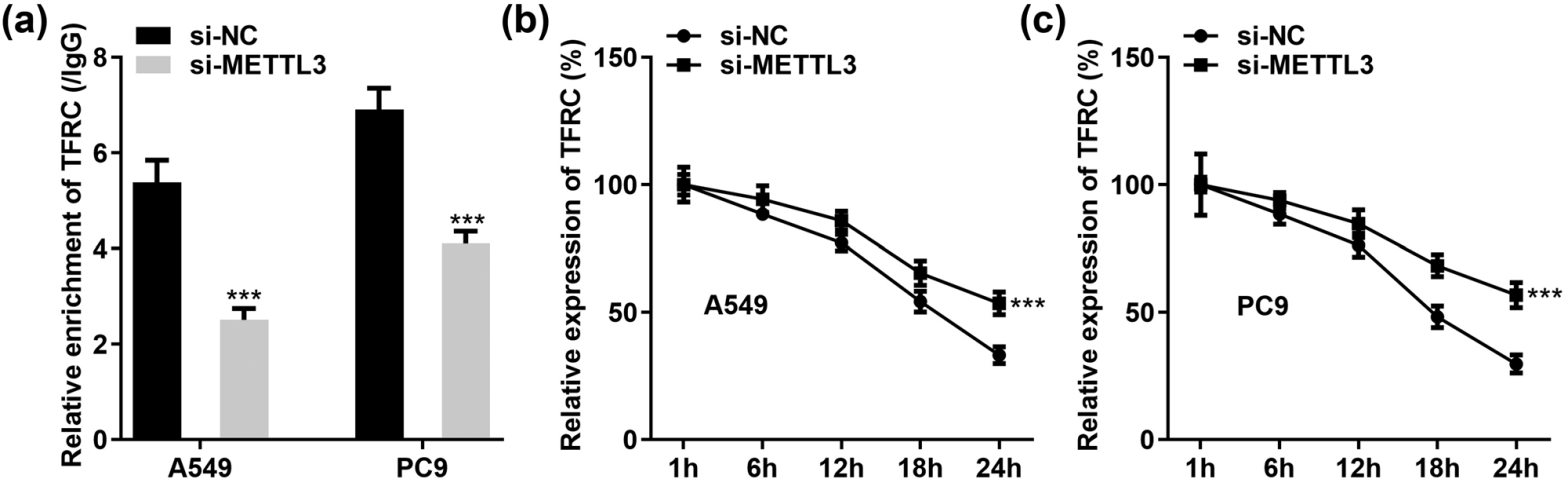
METTL3 silencing enhances the stability of TFRC mRNA. (a) Relative enrichment of TFRC in anti-m^6^A was reduced after treatment with si-NC or si-METTL3 in A549 and PC9 cells. (b and c) Half-life of TFRC mRNA in both cells (A549 and PC9) was prolonged after METTL3 silencing. ***P < 0.001.

Previously, we demonstrated that TFRC levels could be upregulated or reduced in response to METTL3. To further dissect the mechanism by which METTL3 regulates TFRC expression, we performed Me-RIP assays in si-METTL3-treated A549 and PC9 cells. As shown in Figure 6a, METTL3 silencing greatly reduced TFRC enrichment in anti-m^6^A. Regulation of mRNA stability is one of the major functions of m^6^A methylation. Therefore, LC cells were treated with actinomycin D to examine the regulation of TFRC mRNA stability by METTL3. The results presented that METTL3 knockdown effectively prolonged the half-life of TFRC proteins in A549 and PC9 cells (Figure 6b and c). Our study further constructed a correlation between m^6^A methylation and TFRC expression and suggested that METTL3 might hinder TFRC expression in cells by reducing its protein stability in an m^6^A-dependent manner.

## 4 Discussion

Ferroptosis, a novel form of cell death, is driven by the massive accumulation of iron and lethal ROS. It is a central force regulating the growth and proliferation of cancer cells, including LC [18], gastric cancer [19], hepatocellular carcinoma [20], and colorectal cancer [21]. Besides, exploratory studies have identified that triggering ferroptosis might be a possible therapeutic mechanism to eradicate malignant cells [22]. Emerging evidence suggested that METTL3 made a partially important contribution to ferroptosis in LC and hepatoblastoma [12,13]. In most cases, METTL3 exerted oncogenic or tumor suppressive functions through deposition of m^6^A. In highly lethal colorectal cancers, METTL3 was sufficient to stabilize HK2 and GLUT1 expression in an m^6^A-IGF2BP2/3 manner to accelerate tumor progression [23]. In gastric cancer, the enhancement of ZMYM1 mRNA stability by METTL3 was similarly dependent on its m^6^A catalytic activity, and enrichment of ZMYM1 could initiate the EMT procedure [24]. However, this is not always the case; for example, METTL3 mediated tumor inhibition by enhancing p53 protein stability in human LC, which was independent of m^6^A catalytic activity or downstream protein reader [25]. A similar mechanism was seen in the work of Hua et al. The induction of AXL translation by METTL3 in ovarian cancer was similarly independent of its catalytic activity [26]. Given the different mechanisms of METTL3 action in different diseases, we suspected that it might be a valuable biomarker for identifying cancer-impeded ferroptosis and LC. The mechanisms of METTL3 in ferroptosis of LC have rarely been reported. For example, the enrichment of METTL3 in lung adenocarcinoma activated a decrease in SLC7A11 m^6^A levels, which in turn impeded cell ferroptosis and accelerated tumor progression [12,27]. Our results on METTL3 were consistent with earlier work showing that METTL3 had relatively enriched levels in LC and that loss of METTL3 function increased cell ferroptosis sensitivity rather than resistance. Functional inactivation of METTL3 triggered higher levels of Fe^2+^, MDA, and lipid ROS, consistent with the general profile of ferroptosis. The upregulation of lipid ROS levels by definition comes from intracellular GSH depletion [28], and our data also showed that reduced GSH triggered ferroptosis after loss of METTL3 function. Studies have showed that FTH1 and FTL decrease free iron levels during ferroptosis, while inhibition of FTH1 and FTL effectively elevates ferroptosis rate, suggesting a negative correlation between FTH1 and FTL and ferroptosis [29,30]. In our study, loss of METTL3 stimulated A549 and PC9 cells to reduce FTH1 and FTL proteins, triggering ferroptosis.

We also found strong sensitivity and specificity of METTL3 and TFRC levels for the diagnosis of LC, and a negative correlation between the two. A previous study noted that elevated levels of TFRC expression in cancer cells contributed to increased iron uptake, and that degradation of TFRC was essential to reduce essential elements of ferroptosis (unstable iron) [31]. Logically, increasing TFRC expression in cancer cells was expected to cause an increase in iron loading and promote ferroptosis in malignant cells. To establish and obtain plausible results in A549 and PC9 cells, we tested the effect of co-silencing METTL3 and TFRC in these two cell lines. As expected, after the depletion of METTL3, TFRC silencing made cells resistant to the increase of unstable iron, with correspondingly elevated cell activity and highlighted levels of lipids ROS and MDA. In other words, TFRC downregulation might be a key event in the ferroptotic processes. A study on hepatocellular carcinoma reported that intracellular degradation of TFRC impeded the sensitivity of tumor cells to ferroptosis [20,32]. Furthermore, a study on intracerebral hemorrhage indicated that METTL3 induced the development of ferroptosis by regulating the m^6^A level of TFRC mRNA [33]. This prompted us to perform a study of TFRC m^6^A levels and mRNA stability. We found that in the absence of METTL3, m^6^A levels of TFRC were decreased, while mRNA stability was effectively improved. We hypothesized that METTL3 and its mediated stability of TFRC determined the resistance of LC cells to ferroptosis, and that this effect is m^6^A-dependent. The selective mechanism of METTL3 on its direct regulatory targets needs to be further elucidated in the future.

## 5 Conclusion

In conclusion, we demonstrated that METTL3 was overexpressed in primary LC and exhibited specificity and sensitivity in diagnosing LC. METTL3 silencing significantly increased the sensitivity of LC A549 and PC9 cells to ferroptosis, whereas overexpression of METTL3 presented the opposite effect. Further studies showed that METTL3-mediated ferroptosis resulted from its inverse regulation of TFRC expression. In summary, we suggested that an appropriate reduction of METTL3 might be beneficial for LC patients, as it was an effective strategy to sensitize tumors to ferroptosis.
